# A Porous and Conductive Graphite Nanonetwork Forming on the Surface of KCu_7_S_4_ for Energy Storage

**DOI:** 10.3389/fchem.2018.00555

**Published:** 2018-11-21

**Authors:** Wei-Xia Shen, Jun-Min Xu, Shu-Ge Dai, Zhuang-Fei Zhang

**Affiliations:** Key Laboratory of Material Physics of Ministry of Education, Zhengzhou University, Zhengzhou, China

**Keywords:** flexible, porous, graphite nanonetwork, KCu_7_S_4_ nanowires, supercapacitor

## Abstract

A flexible all-solid-state supercapacitor is fabricated by building a layer of porous and conductive nanonetwork on the surface of KCu_7_S_4_ nanowires supported on the carbon fiber fabric, where the porous and conductive nanonetwork is assembled by graphite nanoparticles. This porous graphite layer plays a key role in providing ion diffusion channels to access the KCu_7_S_4_ through the pores for electrochemical reactions and forming electron transport pathways from the graphite network to the electronic collector of the carbon fiber fabric. This flexible supercapacitor exhibits excellent electrochemical performance with high specific capacitance of 408 F g^−1^ at a current density of 0.5 A g^−1^ and high energy density of 36 Wh kg^−1^ at a power density of 201 W kg^−1^. Moreover, it is cost-effective, easy to scale up and environmentally friendly with high flexibility. Our investigation demonstrates that such a porous and conductive nanonetwork could be used to improve the charge storage efficiency for a wide range of electrode materials.

## Introduction

Nowadays, it is a great challenge to develop supercapacitors (SCs) with flexibility, lightweight and high electrochemical performance. In general, the quality of the SCs strongly depends on the design of an appropriate configuration and the innovation of electrode materials (Niu et al., 2013). For the traditional electrodes in SCs, carbonaceous materials (activated carbon, graphite, carbon nanotubes, and graphene) can offer very high power density and excellent cycling ability (Niu et al., 2017; Du et al., 2018; Liu et al., 2018). However, the energy density of carbon-based materials is still too low to meet the requirement for SCs in practical applications (Lu et al., 2014; Guan et al., 2015; Wang et al., 2016; Xia et al., 2017; Dai et al., 2018). Compared with carbon-based SCs, transition-metal oxides/sulfides have attracted particular attention since they could offer much higher energy density by Faradaic reactions (Augustyn et al., 2014; Simon et al., 2014; Dai et al., 2017; Qu et al., 2017; Xu et al., 2017; Zhang et al., 2018a). However, they usually suffer from low electrical conductivity, poor rate performance and limited cycling stability (Liu et al., 2010; Xia et al., 2014; Dai et al., 2016a; Jiang et al., 2018). To overcome the accumulation of produced charges on the surface of pseudo-capacitor material which could not successfully reach electron collector, the design of hybrid structure electrodes is an efficient way for SCs with excellent electrochemical performance (Chang et al., 2012; Qu et al., 2018a,b; Zhang et al., 2018b; Zheng et al., 2018a).

Recently, transition-metal oxides are emerging as promising electrode materials for energy storage devices, such as RuO_2_, MnO_2_, NiO, Fe_2_O_3_, WO_3_, V_2_O_5_ (Xue et al., 2011; Dang et al., 2018; Zheng et al., 2018b). Among them, manganese oxides have been widely studied as electrode materials for SCs due to their high theoretical capacitance, low-cost, environmentally friendliness and natural abundance. The α-MnO_2_ is constructed from double chains of octahedral [MnO_6_] structure with 2 × 2 and 1 × 1 tunnels, which is beneficial for Li^+^ transportation (Park et al., 2007; Reddy et al., 2009). However, its actual capability is often much lower than the theoretical value owing to its low electronic conductivity. Besides, it also displays poor capacity retention and large volume change during Li^+^ insertion/extraction (Wang et al., 2014a). Similar to the crystal structure of α-MnO_2_, the KCu_7_S_4_ has one-dimensional double tunnels along c axis, which is composed of a three-dimensional Cu-S framework that contains pseudo-one-dimensional channels in which K ions reside in the channels (Hwu et al., 1998; Dai et al., 2013, 2014a). Compared with a-MnO_2_, the KCu_7_S_4_ exhibits greater conductivity and capacity retention, which is one of the most promising electrode materials for energy storage (Dai et al., 2013, 2014a; Guo et al., 2016). Moreover, the KCu_7_S_4_ has significant advantages, such as large surface area, low-cost, easy synthesis, and environmentally friendliness. To improve the performance, many researchers have focused on the surface modification of the micro/nano electrode materials, such as Au nanoparticles coated WO_3−x_ NWs (Lu et al., 2012), graphene quantum dots coated VO_2_ arrays (Chao et al., 2015), CNTs decorated MoO_3_ (Yang et al., 2014a). It is an effective way to enhance the electrical conductivity of the electrode materials, which improves the ion diffusion kinetics and electron transport by coating of nanostructured conductive layer. Herein, we design a porous and conductive nanonetwork by coating graphite nanoparticles on the surface of KCu_7_S_4_ nanowires, which not only ensures the multichannel diffusion of electrolyte ions insert the KCu_7_S_4_ material, but also improves the electron transportation. It is no doubt that this porous and conductive nanonetwork structure will attract more attention in the design of the electrodes for SCs.

Currently, the fabricated electrodes based on KCu_7_S_4_ materials are too rigid and bulky, which could not meet the practical requirements for flexible and wearable electronic devices (Dai et al., 2014b, 2015). Therefore, the exploration of flexible, lightweight, or even wearable SCs based on the KCu_7_S_4_ materials will be interesting work. Recently, carbon fiber fabric (CFF) attracts many people's interest because of its unique characteristics, such as low corrosion resistance, low thermal expansion coefficient and excellent flexibility. Moreover, all-solid-state supercapacitors based on CFF can be easily bent or twisted, which could meet the requirements for flexible and wearable electronic devices (Yuan et al., 2012).

In this work, we report a highly flexible all-solid-state SC based on a layer of porous and conductive graphite nanonetwork coated on the surface of KCu_7_S_4_ nanowires, which is supported on a carbon fiber fabric (GN/KCu_7_S_4_/CFF). The GN/KCu_7_S_4_/CFF SC exhibits great electrochemical performance with the highest specific capacitance of 408 F g^−1^ and the highest energy density of 36 Wh kg^−1^ at a power density of 201 W kg^−1^. The enhanced capacity attributed to the porous and conductive nanonetwork on the surface of the KCu_7_S_4_ nanowires, which provides rich ion diffusion channels to access the KCu_7_S_4_, and shortens the electron transmission paths through the graphite network to the electronic collector of CFF. This work demonstrates that the porous and highly conductive graphite nanonetwork could be used to improve the charge storage for a wide range of electrode materials, revealing a promising application in the flexible energy-storage devices.

## Experimental section

### Preparation of GN/KCu_7_S_4_/CFF electrode

Carbon fiber fabric (Shanghai Lishuo Composite Material Technology Company) and the graphite ink (from Hero, Shanghai Ink Factory in China) were used as purchased. First, 1 mmol of CuCl_2_·2H_2_O, 2.5 mmol of S, and 53 mmol of KOH were dissolved in deionized water (10 mL) in the Teflon containers, followed by addition of 2 mL of hydrazine monohydrate. Then the mixed solution was retained at 150°C for 12 h. After cooled down to room temperature, the product was rinsed with ultrapure water, and dried under vacuum at 60°C overnight. The GN/KCu_7_S_4_/CFF was made as follows: 100 mg of as-prepared KCu_7_S_4_ nanowires was first dispersed in ultrapure water (10 mL). Then the graphite ink was dropped into the KCu_7_S_4_ solution (the ratio of ink to water is 1:10) under magnetic stirring for 24 h at 95°C. Finally, the mixture was filtered on the CFF to obtain the GN/KCu_7_S_4_/CFF, where free nanoparticles were removed through the pores of the CFF. The product was put into oven for 2 h at 60°C for drying.

### Fabrication of all-solid-state supercapacitor

The separator (Whatman 8 μm filter paper) covered with a layer of PVA-LiCl gel as a solid electrolyte on both sides and, sandwiched between the two pieces of the GN/KCu_7_S_4_/CFF electrodes to form a two electrode device. The detailed fabrication process of the electrode was reported in our previous work (Javed et al., 2015). Here, the mass loading on the carbon fiber fabric is about 2 mg cm^−2^ and the working area of each electrode is 4 cm × 1.5 cm.

### Characterization and the electrochemical measurements

The morphology, chemical composition, and the structure of the products were observed by X-ray diffraction (XRD) analysis (XRD, PA National X′ Pert Pro with Cu Kα radiation). The microstructure and morphology of NC nanomaterials were characterized using field emission scanning electron microscopy (Zeiss, sigma300) and high-resolution transmission electron microscopy (HRTEM, JEOL, JEM-2100) with energy dispersive X-ray spectrometry (EDS). The nitrogen adsorption-desorption isotherm measurement of the sample was performed using a ASAP2420-4MP. The specific surface area was obtained by the Brunauer-Emmett-Teller (BET) method. The electrochemical measurement was conducted with an electrochemical workstation (CHI 760D). X-ray photoelectron spectrometer (XPS) analysis was performed on an ESCA Lab MKII using Mg Ka as the exciting source.

## Results and discussion

**Figure 1:**
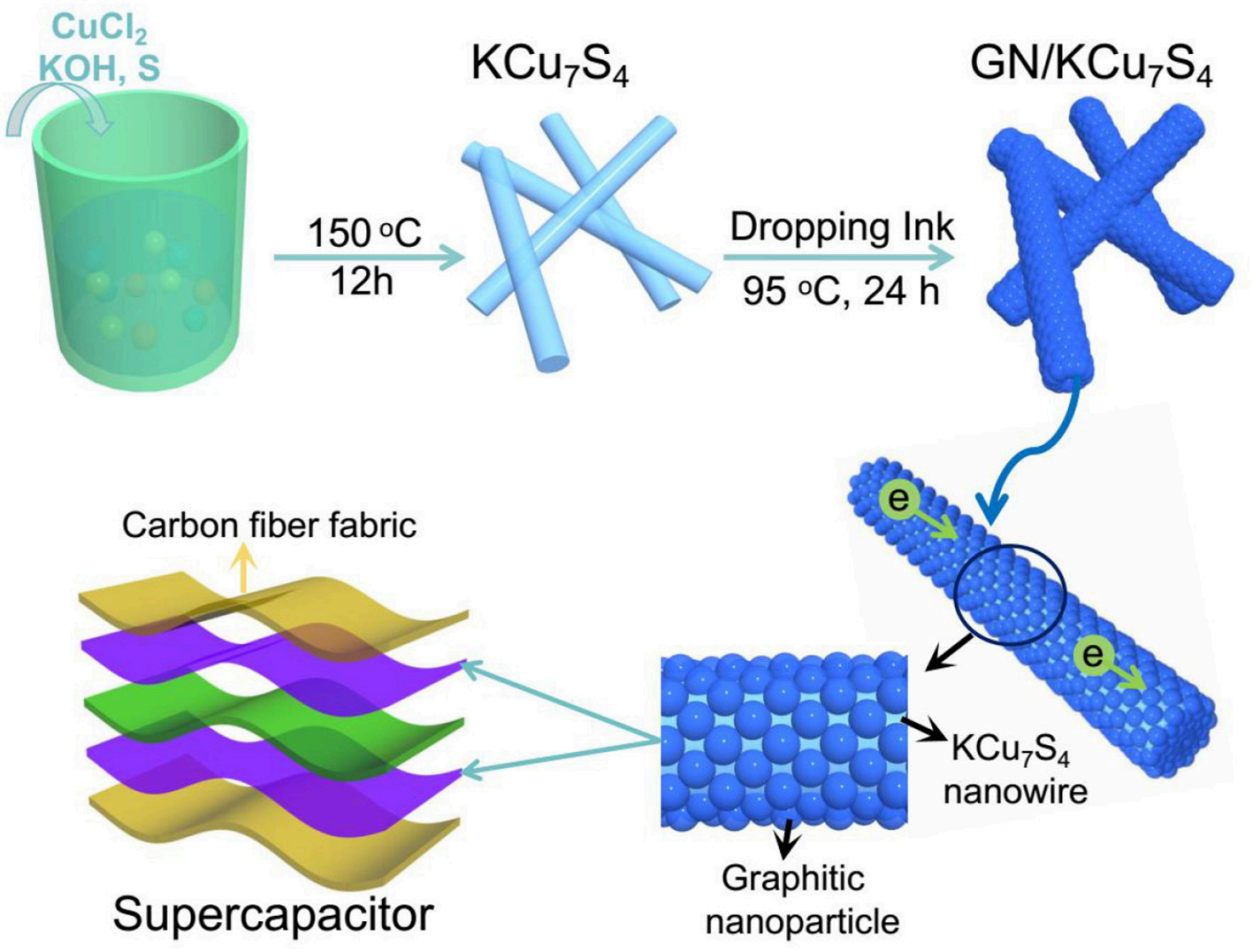
Schematic diagram of preparing of GN/KCu_7_S_4_ nanowires and the fabrication of flexible GN/KCu_7_S_4_/CFF SC.

**Figure 2:**
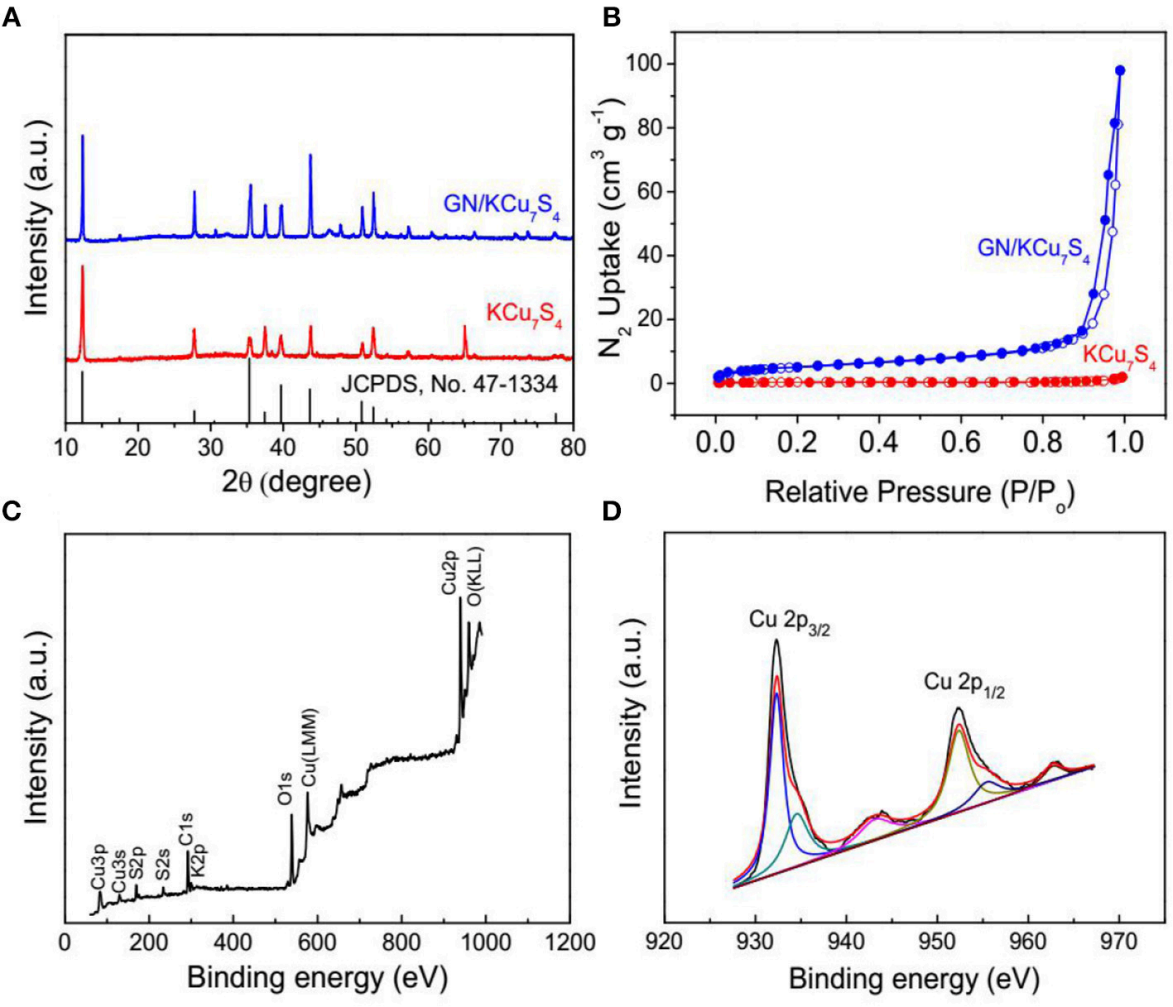
**(A)** XRD patterns of KCu_7_S_4_ and GN/KCu_7_S_4_ samples. **(B)** The nitrogen adsorption-desorption isotherms of as-prepared KCu_7_S_4_ and GN/KCu_7_S_4_ samples. **(C)** XPS spectra of as-prepared KCu_7_S_4_ sample, and **(D)** Cu 2p XPS spectra of as-prepared KCu_7_S_4_ sample.

**Figure 3:**
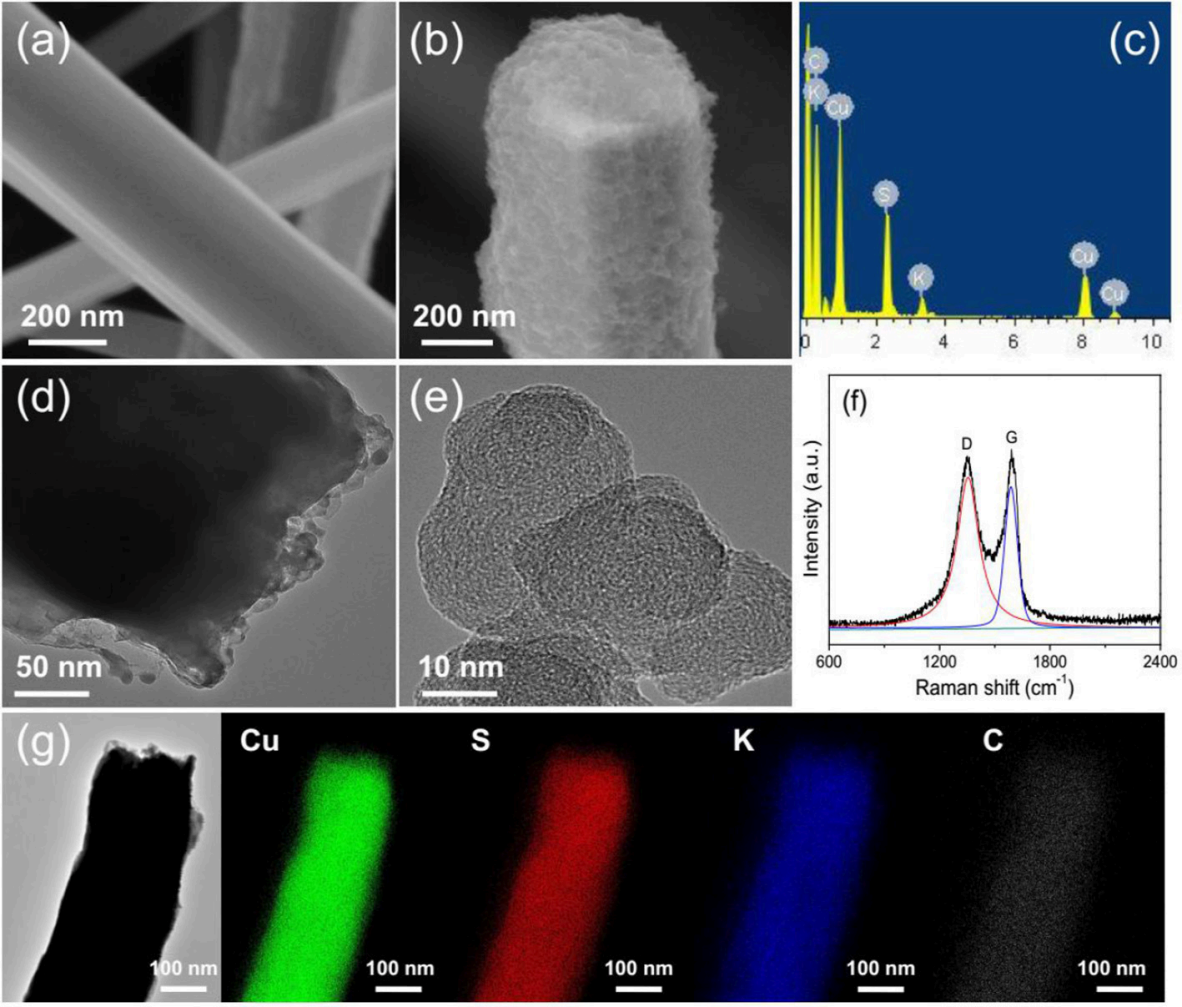
**(a)** SEM image of the KCu_7_S_4_ nanowires. **(b)** SEM images of the GN/KCu_7_S_4_ nanowires. **(c)** EDS of a single GN/KCu_7_S_4_ nanowire. **(d)** TEM images of the GN/KCu_7_S_4_ nanowires and **(e)** graphite nanoparticle. **(f)** Raman spectrum of the graphite ink. **(g)** TEM-EDX mapping images of GN/KCu_7_S_4_ nanowire.

Figure 1 shows the schematic diagram of preparing of GN/KCu_7_S_4_ nanowires and the fabrication of flexible GN/KCu_7_S_4_/CFF SC, respectively. The X-ray diffraction of KCu_7_S_4_ and GN/KCu_7_S_4_ nanowires indicate that the samples are well crystallized (Figure 2A). All the diffraction peaks can be unambiguously assigned to tetragonal KCu_7_S_4_ structure. To understand the porosity and surface area of as-prepared samples, N_2_ adsorption-desorption isotherms of KCu_7_S_4_ and GN/KCu_7_S_4_ conducted at 77.350 K were investigated and are displayed in Figure 2B. Through BET analysis, the surface areas of KCu_7_S_4_ and GN/KCu_7_S_4_ samples were identified as 1 m^2^g^−1^ and 18.6 m^2^g^−1^, respectively. To identify the chemical states of Cu element in the samples, the XPS survey spectrum of the KCu_7_S_4_ nanowires and high-resolution XPS spectrum of Cu 2p were also conducted (Figures 2C,D). It consists of two binding energy of Cu 2p_3/2_ and Cu 2p_1/2_ peaks at 932.3 and 952.2 eV, respectively, which are in agreement with the previous reports (Colleen and McShane, 2012; Wang et al., 2013). Scanning electron microscopy (SEM) images of as-prepared KCu_7_S_4_ and GN/KCu_7_S_4_ samples are shown in Figure 3, Figure S1. The KCu_7_S_4_ nanowires have a diameter of 200–500 nm and length up to 110 μm. The enlarged image (Figure 3b) of GN/KCu_7_S_4_ nanowires clearly indicates that the KCu_7_S_4_ nanowires were coated with graphite nanoparticles with high homogeneity. For further confirmation, the EDS of a single GN/KCu_7_S_4_ nanowire is presented in Figure 3c, revealing the main compositions of C, K, Cu, and S. This good composite nanostructure was also further confirmed by transmission electron microscopy (TEM) analysis, as shown in Figures 3d,e. In order to explore the composition of the graphite ink and GN/KCu_7_S_4_, we also carried out a Raman test and the results are presented in Figure 3f, Figure S2. The G and D peaks are clearly observed at 1355 cm^−1^ (attributed to the disordered carbonaceous component) and 1585 cm^−1^ (attributed to the ordered graphitic component), respectively, which exhibits that the active component in graphite ink is mainly graphitic carbon (Cai et al., 2012; Dai et al., 2014c). The peak at 472 cm^−1^ corresponds to the KCu_7_S_4_ (Figure S2). Moreover, the TEM-EDX elemental mapping of the GN/KCu_7_S_4_ reveals a relatively uniform distribution of K, Cu, S, and C elements over the nanowire, which indicates the KCu_7_S_4_ nanowires were well wrapped by the graphite nanoparticles. Owing to the strong adhesion of the graphite nanoparticles bounded together to form a porous nanonetwork structure on the surface of the KCu_7_S_4_ nanowires, the nanonetwork can provide efficient ion diffusion multichannels to access the KCu_7_S_4_ and shorten the electron transport pathways to the electronic collector of CFF (Fu et al., 2012; Dai et al., 2016b).

**Figure 4:**
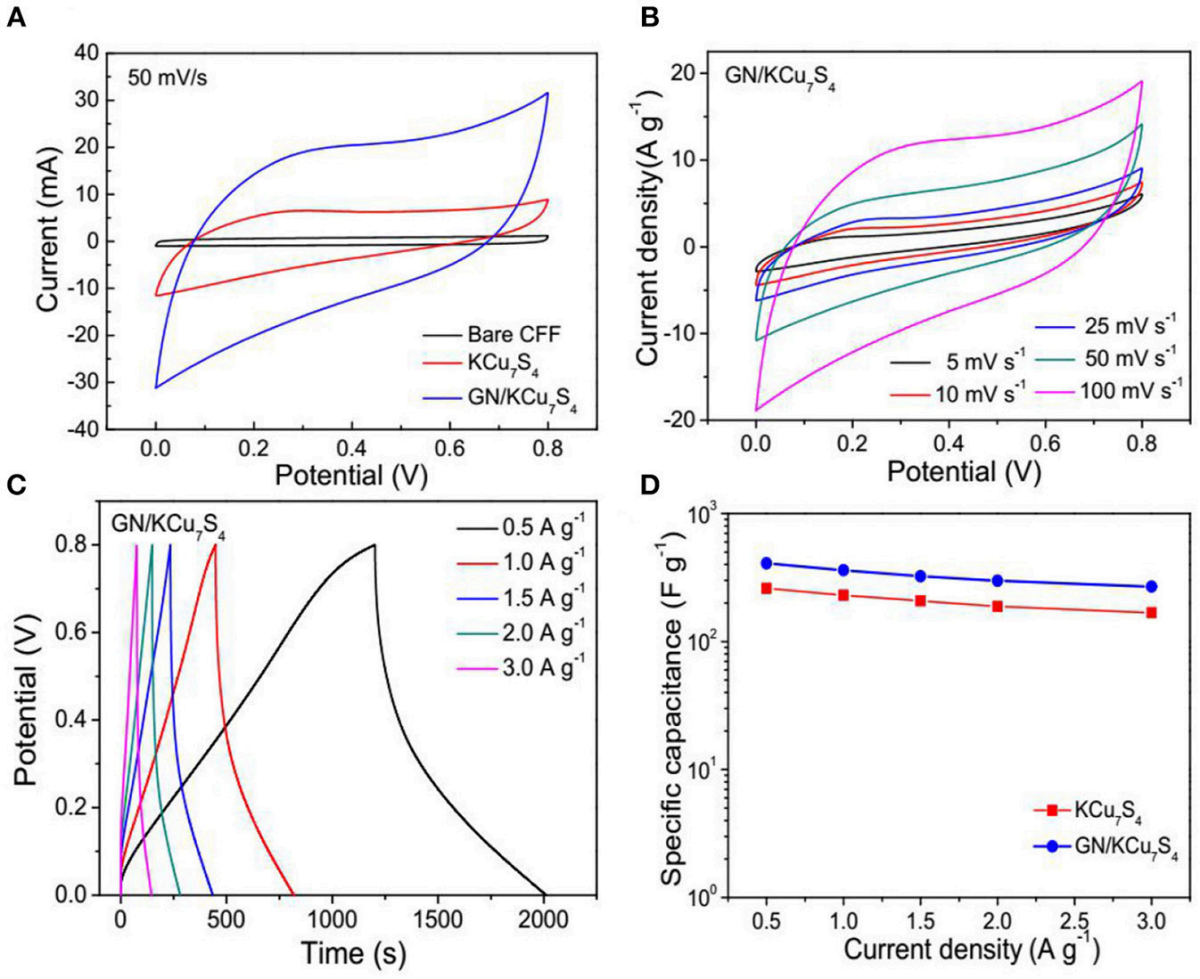
**(A)** CV curves of bare CFF, KCu_7_S_4_ /CFF and GN/KCu_7_S_4_/CFF SCs at a scan rate of 100 mV/s. **(B)** CV curves of the GN/KCu_7_S_4_/CFF SC at different scan rates. **(C)** Galvanostatic charge-discharge curves of the GN/KCu_7_S_4_/CFF SC at various current densities. **(D)** Specific capacitances of the KCu_7_S_4_ /CFF and GN/KCu_7_S_4_/CFF SCs at different current densities.

The electrochemical performance of the supercapacitors based on the KCu_7_S_4_/CFF and GN/KCu_7_S_4_/CFF are characterized by using cyclic voltammetry (CV), galvanostatic charge-discharge (GCD) cycling and electrochemical impedance spectroscopy (EIS), respectively. Figure 4A shows the CV curves of the bare CFF, KCu_7_S_4_, and /GN/KCu_7_S_4_/CFF based SCs at a constant scan rate of 100 m V/s. It is note that the GN/KCu_7_S_4_/CFF SC shows a higher capacitance behavior as compared with others. Figure 4B exhibits the CV curves of the GN/KCu_7_S_4_/CFF SC at different scan rates in potential windows from 0 to 0.8 V. All the CV curves exhibit an approximate shape with slight variations, even at a scan rate of 100 m V/s, revealing the good capacitive behavior of the GN/KCu_7_S_4_/CFF electrodes. The CV curves of KCu_7_S_4_ SC at different scan rates were also collected and is shown in Figure S3A. The galvanostatic charge-discharge curves of the GN/KCu_7_S_4_/CFF SC at various current densities in potential windows from 0 to 0.8 V (Figure 4C) exhibit good linear and almost symmetrical voltage-time profiles with small *IR* drops, indicating high output power of the GN/KCu_7_S_4_/CFF SC. The corresponding galvanostatic charge-discharge curves of the KCu_7_S_4_/CFF SC at various current densities are shown in Figure S3B. The specific capacitances of KCu_7_S_4_/CFF and GN/KCu_7_S_4_/CFF SCs were calculated by the mass loading of KCu_7_S_4_ and GN/KCu_7_S_4_ NWs on the CFFs, respectively, and the results are shown in Figure 4D. The maximum specific capacitance of 408 F g^−1^ at a current density of 0.5 A g^−1^ for the GN/KCu_7_S_4_/CFF SC was calculated, which is two times higher than that of KCu_7_S_4_/CFF SC (167 F g^−1^). The enhanced electrochemical performance of the GN/KCu_7_S_4_/CFF electrodes benefits from the following facts. First, the nanonetwork assembled by the graphite nanoparticles on the surface of KCu_7_S_4_ nanowires improves the conductivity of the KCu_7_S_4_ nanowires, which greatly increases the electron transmission rate. Secondly, these nanoparticles aggregated together to form a porous structure on the surface of the KCu_7_S_4_ nanowires, which provides rich channels for ions to access to electroactive sites for fast and reversible redox reactions (Guan et al., 2015). The specific capacitance of the GN/KCu_7_S_4_/CFF SC in this work is higher than that of the previously reported for the hybrid SCs, such as 80.8 F g^−1^ at 0.5 A g^−1^ for the GNS/αMWCNT@PDAA SC (Sun et al., 2015), 56 F g^−1^ at 0.58 A g^−1^ for the MSCS-O SC (Kim et al., 2015), 156 F g^−1^ at 0.5 A g^−1^ for the PG-paper SC (Shu et al., 2015), and 189 F g^−1^ at 0.5 A g^−1^ for the FeMnO_3_/RGO SC (Li et al., 2014). These results indicate that the electrochemical performance of the KCu_7_S_4_ nanowires is improved by the successful coating of the graphite nanoparticles and this method can also be applied for other metal sulfides.

The EIS is measured in the frequency from 100 kHz to 1 Hz, and the Nyquist impedance plots of the KCu_7_S_4_/CFF and GN/KCu_7_S_4_/CFF SCs are shown in Figure S4A. In the high frequency range, the intercepts of the Nyquist curves on the real axis are about 2.43 Ω and 2.18 Ω for the KCu_7_S_4_/CFF and GN/KCu_7_S_4_/CFF SCs, respectively, indicating better conductivity after coating the graphite nanoparticles. A smaller arc is observed for the GN/KCu_7_S_4_/CFF SC, which demonstrates an enhanced ion accessibility of the GN/KCu_7_S_4_ nanowires compared with that of KCu_7_S_4_ nanowires, due to the highly porous network structure. The Nyquist plots show almost a vertical line in the low frequency, indicating an excellent capacitive behavior of SC. To obtain more detailed information, the dependence of the phase angle on the frequency for the KCu_7_S_4_/CFF and GN/KCu_7_S_4_/CFF SCs are shown in Figure S4B. The relaxation time τ_0_(_τ_0_ = 1/*f*_0__) evaluated from the frequency at 45° impedance phase angle is 0.09 s for the GN/KCu_7_S_4_/CFF, which is shorter than that of the KCu_7_S_4_/CFF (0.14 s), revealing larger power response of the GN/KCu_7_S_4_/CFF SC (Liu et al., 2015).

**Figure 5:**
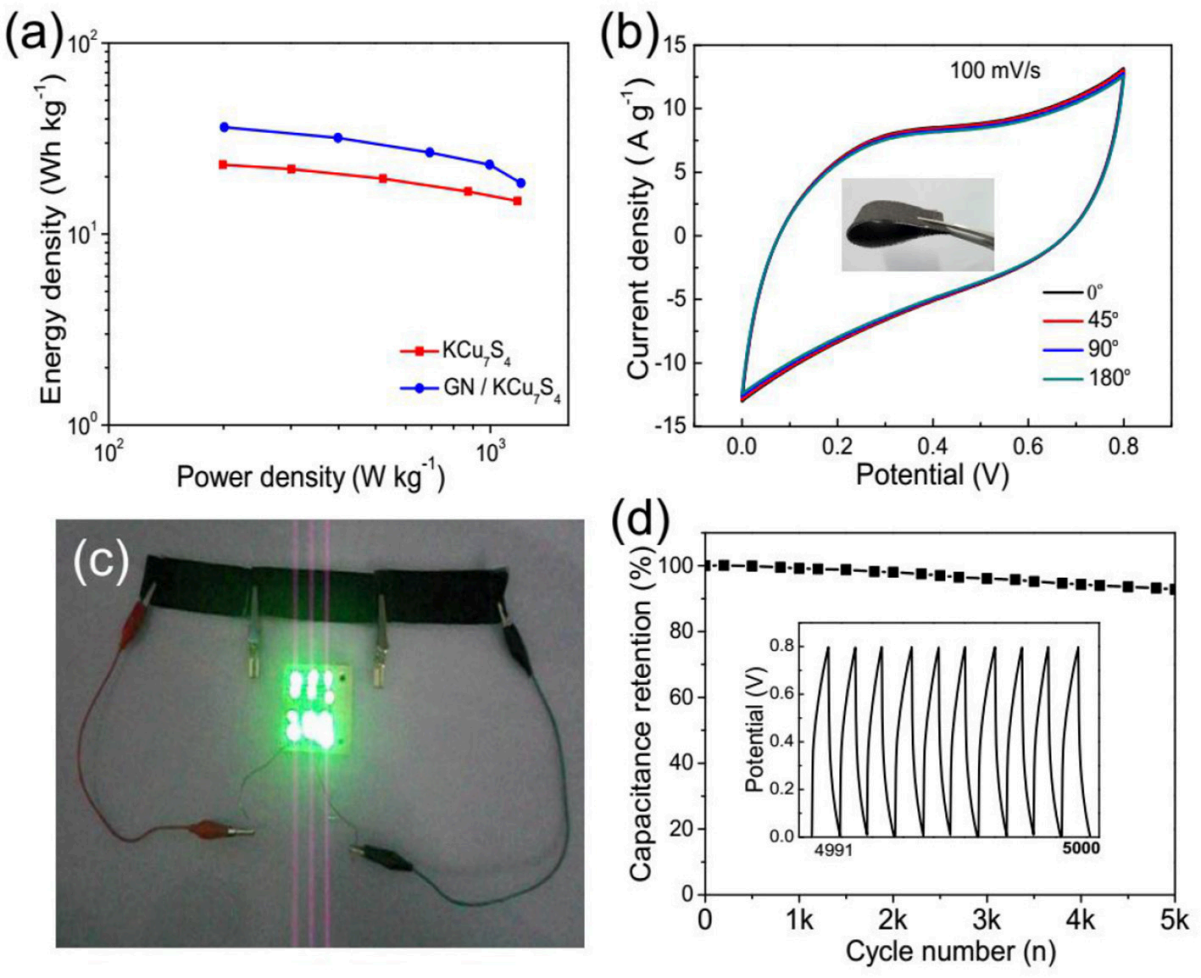
**(a)** Ragone plots of the prepared KCu_7_S_4_ /CFF and GN/KCu_7_S_4_/CFF SCs. **(b)** CV curves of the GN/KCu_7_S_4_/CFF SC at different curvatures of 0^o^, 45^o^, 90^o^, and 180^o^. **(c)** Optical photograph of 12 commercialized LEDs lighted by three GN/KCu_7_S_4_/CFF SCs connected in series. **(d)** Cycling life of the GN/KCu_7_S_4_/CFF SC.

Energy density (*E*) and power density (*P*) are two important parameters for evaluating the electrochemical performance of SCs (Lu et al., 2012). The energy density viruses the average power density is calculated from the charge-discharge curves (Figure 5a), which are estimated according to the following equations (Dai et al., 2014c).

(1) $$\begin{array}{r} E=\frac{CV^{2}}{2M} \end{array}$$

(2) $$\begin{array}{r} P=\frac{E}{t} \end{array}$$

where *C, M, V, and t* are the total capacitance of the device, effective mass of the electrode, voltage and the discharge time, respectively. The highest energy density of the GN/KCu_7_S_4_/CFF SC is 36 Wh kg^−1^ at a power density of 201 W kg^−1^, which is higher than that of KCu_7_S_4_/CFF SC with the energy density of 14 Wh kg^−1^ at a power density of 190 W kg^−1^. The maximum energy density of the GN/KCu_7_S_4_/CFF SC is higher than those previously reported, such as 6.3 Wh kg^−1^ for the WL-MnO_2_ SC (Yang et al., 2014b), 17 Wh kg^−1^ for the MnFe_2_O_4_/graphene/polyaniline SC (Sankar and Selvan, 2015), 12.3 Wh kg^−1^ for the MnO_2_@KCu_7_S_4_ hybrid SC (Wang et al., 2014c), 22 Wh kg^−1^ for the CoOH//VN SC (Wang et al., 2014b), and 1.46 Wh kg^−1^ for the Al-doped α-MnO_2_ SC (Hu et al., 2015).

For efficient energy storage devices, flexible, lightweight, and portable electronic devices are desired in practical applications. Figure 5b displays the high flexibility of as-prepared GN/KCu_7_S_4_/CFF SC, and it can be folded and twisted without destroying its physical structure. Moreover, the CV curves of the GN/KCu_7_S_4_/CFF SC hardly change under different bending angles, indicating its good flexibility. For practical applications, it is necessary to connect SCs in series and/or in parallel to increase the operating voltage and/or current in some situations (Yuan et al., 2013). Figure 5c shows three GN/KCu_7_S_4_/CFF SCs connected in series can light 12 commercial light-emitting diodes (LEDs) for about 5 min after charging at 12 A g^−1^ for 50 s (for detailed information, see Supporting Information). The excellent properties of the flexible GN/KCu_7_S_4_/CFF SC reveal a potential application in superior storage devices. In addition, the GN/KCu_7_S_4_/CFF SC exhibits a long-term cycling stability between 0 and 0.8 V at a current density of 2 A g^−1^ and keeps 90% of its initial capacitance after 5,000 cycles (Figure 5d), revealing its good cycling life.

## Conclusion

In summary, we have successfully designed a porous and highly conductive nanonetwork structure electrode by coating graphite nanoparticles on the surface of the KCu_7_S_4_ nanowires. Such a porous nanonetwork not only facilitates the diffusion of the electrolyte ions into the pseudocapacitive material, but also improved the electron transmission, which greatly enhance the charge storage efficiency. Moreover, a highly flexible all-solid-state hybrid SC based on the GN/KCu_7_S_4_ nanowires is fabricated, which shows excellent electrochemical properties, including the high specific capacitance (408 F g^−1^), high energy density (36 Wh kg^−1^), and good cyclic stability. All the results indicate that such porous and highly conductive nanonetwork forming on nanostructured pseudocapacitive materials could improve the charge storage efficiency of supercapacitors.

## Author contributions

W-XS carried out the material preparation, electrochemical test, and analyzed the XRD, SEM, TEM, and Raman analysis. S-GD wrote the paper and J-MX discussed the results and revised the manuscript. Z-FZ attained the main financial support for the research and supervised all the experiments.

### Conflict of interest statement

The authors declare that the research was conducted in the absence of any commercial or financial relationships that could be construed as a potential conflict of interest.
