# Supplementary material for: A Porous and Conductive Graphite Nanonetwork Forming on the Surface of KCu7S4 for Energy Storage
Source: Front Chem. 2018 Nov 21;6:555. doi: 10.3389/fchem.2018.00555 (PMC6258969; doi:10.3389/fchem.2018.00555)
Supplement: Supplementary file 2 [file Data_Sheet_1.PDF]

Supporting Information

A porous and conductive graphite nanonetwork forming on the surface of KCu_7S_4 for energy storage

Wei-Xia Shen, Jun-Min Xu, Shu-Ge Dai, Zhuang-Fei Zhang**

Key Laboratory of Material Physics of Ministry of Education, Zhengzhou University, Zhengzhou 450052, China;

*Corresponding author

School of Physics and Engineering, and Key Laboratory of Material Physics of Ministry of Education, Zhengzhou University, Zhengzhou 450052, PR China

E-mail addresses: shugedai@zzu.edu.cn (S. Dai); zhangzf@zzu.edu.cn (Z. Zhang)

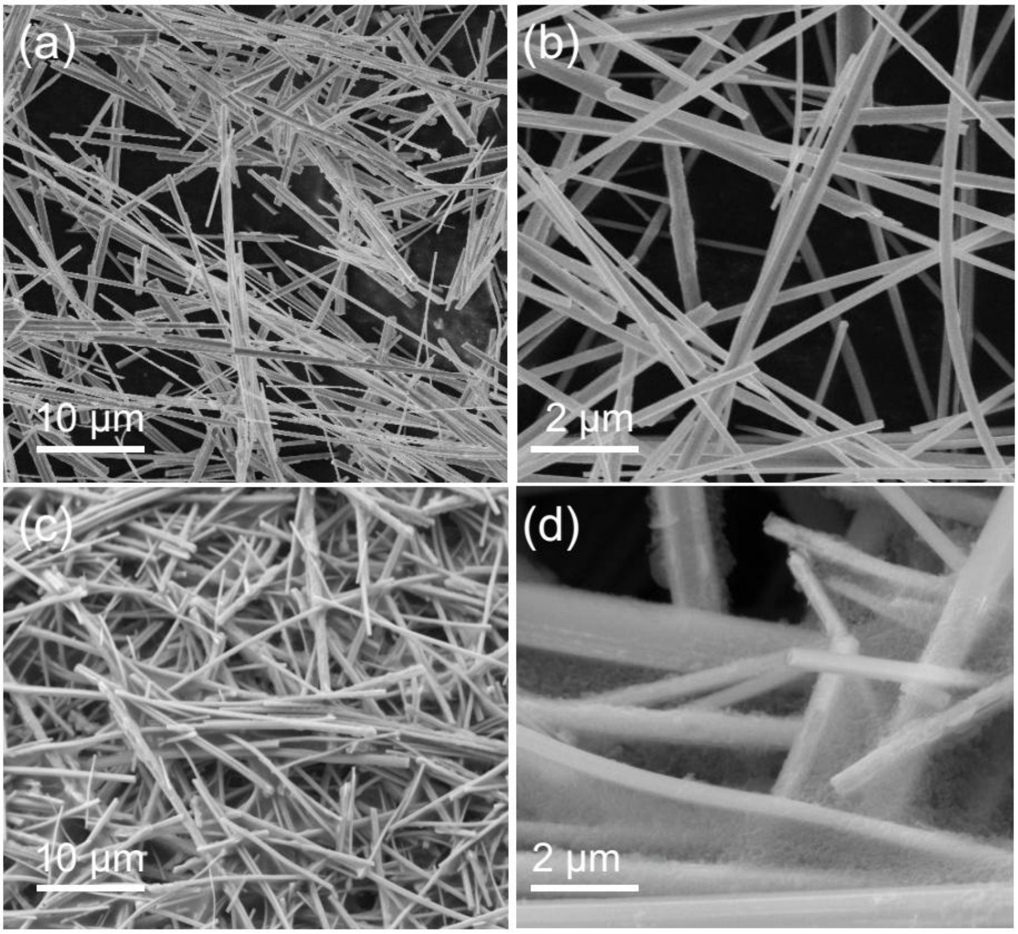

FIGURE. S1 (a, b) SEM images of KCu_7S_4 nanowires. (c,d) SEM images of GN/KCu_7S_4 nanocomposites.

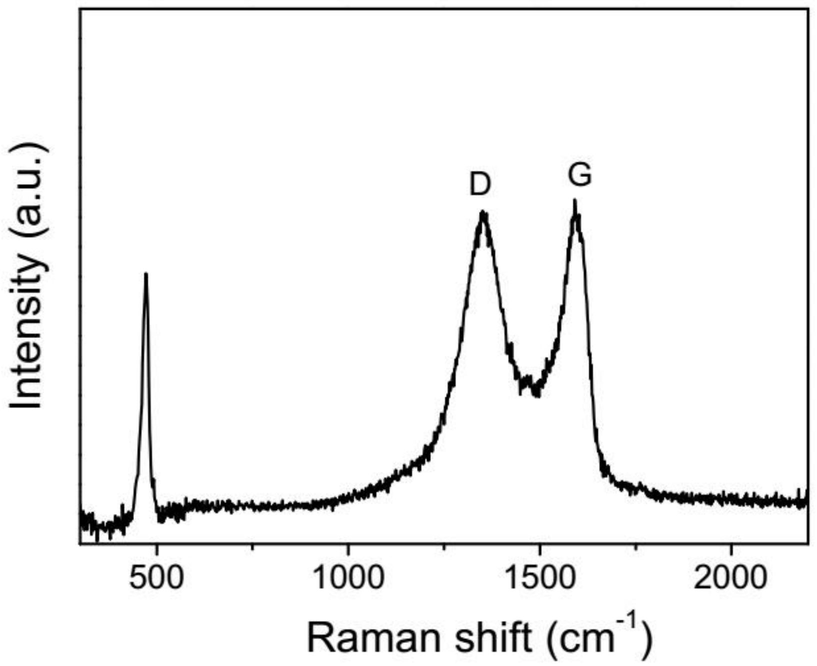

FIGURE. S2 Raman spectrum of the GN/KCu_7S_4 nanowires

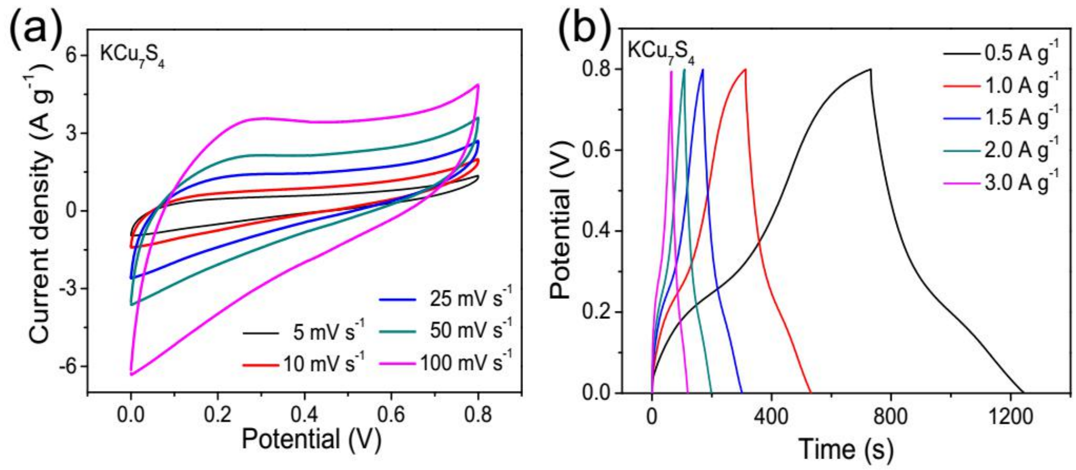

FIGURE. S3 (a) CV curves of KCu_7S_4 /CFF SCs at different scan rates. (b) Galvanostatic charge-discharge curves of the KCu_7S_4/CFF SC at various current densities. .

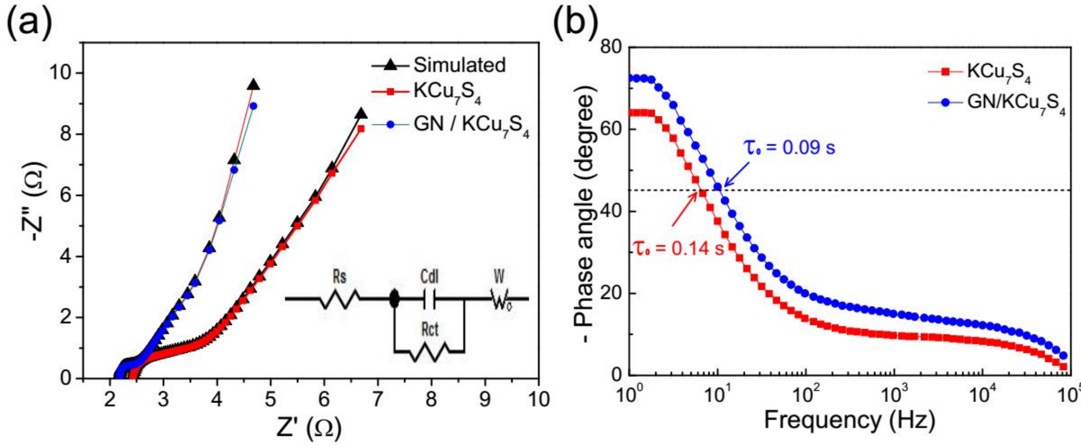

FIGURE. S4 (a) Nyquist plots, (b) Bode plots of the prepared KCu_7S_4/CFF and GN/KCu_7S_4/CFF SCs.

VIDEO. Demonstration of the devices powering the LEDs in practical application.
